# Real-World Effectiveness and Safety of Isavuconazole Versus Amphotericin B for Patients with Invasive Mucormycosis

**DOI:** 10.3390/microorganisms13010055

**Published:** 2025-01-01

**Authors:** Jiayuan Qin, Hongxia Bi, Guangmin Tang, Xinyao Liu, Junyan Qu, Xiaoju Lv, Yanbin Liu

**Affiliations:** 1 Center of Infectious Diseases, West China Hospital, Sichuan University, Guoxuexiang 37, Chengdu 610041, China; jiayuanqin@wchscu.cn (J.Q.); doctorbi619@163.com (H.B.); guangmintang@outlook.com (G.T.); qujunyan15647@wchscu.cn (J.Q.); lvxj@scu.edu.cn (X.L.); 2 State Key Laboratory of Biotherapy, Division of Infectious Diseases, Chengdu 610041, China; 3 Center for Pathogen Research, West China Hospital, Sichuan University, Chengdu 610041, China; katrina_9637@163.com

**Keywords:** isavuconazole, amphotericin B, invasive mucormycosis, effectiveness, safety, real-world

## Abstract

Background: Invasive mucormycosis (IM) poses a substantial morbidity and mortality burden among immunocompromised patients. Objectives: We aim to compare the real-world effectiveness and safety of isavuconazole with those of amphotericin B in patients with IM. Patients and methods: In this observational cohort study, we enrolled patients who were diagnosed with IM and treated with either isavuconazole or amphotericin B. Results: A total of 106 patients met the study criteria. Of these, 47 received isavuconazole, and 59 received amphotericin B as the primary treatment. The two cohorts had similar baseline characteristics, including a history of malignancy, use of immunosuppressants, infection sites, and pathogens. The amphotericin B group demonstrated a significantly greater incidence of renal disorders (*p* < 0.001) and hypokalemia (*p* < 0.001) than the isavuconazole group. The proportion of patients who received salvage therapy was greater in the amphotericin B group than in the isavuconazole group (42% vs. 6%, *p* < 0.001). Eighteen patients in the amphotericin B group discontinued treatment because of adverse events, whereas no patients in the isavuconazole group discontinued treatment because of adverse events. A significant difference in the primary therapeutic response between the isavuconazole and amphotericin B groups was noted (*p* = 0.013), with a higher treatment failure rate in the amphotericin B group (68% vs. 36%, *p* = 0.001). However, there were no significant differences in all-cause mortality or mucormycosis-attributable mortality rates between the two groups. Conclusions: Isavuconazole outperformed amphotericin B as a first-line treatment option for IM in terms of its clinical effectiveness and safety.

## 1. Introduction

Invasive mucormycosis (IM) is a rapidly progressive, angioinvasive fungal infection caused by filamentous fungi of the order *Mucorales*. IM predominantly affects immunocompromised patients, including those with hematologic malignancies and recipients of hematopoietic stem cells or solid organ transplants [1]. Population-based investigations indicate that the global prevalence of IM has increased in recent years, which is correlated with the expanding use of corticosteroids and immunosuppressive therapies, as well as with the global COVID-19 pandemic [2,3,4]. Active population-based laboratory surveillance in the United States estimated the cumulative incidence of IM to be 1.7 cases per million person-years [5]. This figure has steadily increased, and by 2016, the number of hospitalizations associated with mucormycosis had reached 16 per million person–years [6]. The incidence of IM in India has consistently been much higher than the global average, particularly during the COVID-19 pandemic [3,7]. In China, an increasing annual infection rate has also been reported [8]. The prognosis of IM remains poor, with a reported mortality rate ranging from 22% to 38%, depending on the underlying conditions, site of infection, and management strategies [2,9,10,11,12,13]. Furthermore, patients with mucormycosis have significantly longer hospital stays and higher total hospitalization costs than patients without mucormycosis [9,14,15]. IM has become a serious global concern because of its increasing incidence, high mortality rate, and substantial economic burden.

For decades, amphotericin B has been a cornerstone in the treatment of mucormycosis, and it has been advocated for inclusion among the therapeutic arsenal of effective antifungal agents targeting IM [16,17,18,19]. However, its application is constrained by concerns about toxicity and the requirement for intravenous administration, even with liposomal formulations [16,20,21]. Furthermore, amphotericin B and its liposomal formulations are often recommended for lengthy periods (4–6 weeks), extending hospital stays and increasing the risk of central line-associated bloodstream infections [16,20,22]. This lengthy administration is not feasible in resource-limited settings where treatment interruptions are common. This restriction leads to a dilemma in how best to manage IM.

Isavuconazole, a novel second-generation broad-spectrum triazole, received U.S. Food and Drug Administration approval for the treatment of IM in 2015, and it is recommended as a first-line treatment in some guidelines [16,23,24]. Although clinical research suggests that isavuconazole is as effective as amphotericin B and is associated with fewer adverse events [25], real-world data comparing their effectiveness and safety remain scarce. This study aims to address this gap in knowledge by comparing the real-world clinical outcomes and safety profiles of isavuconazole and amphotericin B in patients with IM, thereby providing critical evidence to guide the treatment selection and clinical management of IM.

## 2. Material and Methods

### 2.1. Study Design and Data Collection

This real-world observational cohort study was conducted from January 2022 to June 2024 at a large comprehensive medical center in Western China with 4300 inpatient beds. The authors confirm that the ethical policies of the journal, as noted on the journal’s author guidelines page, have been followed and that appropriate ethical review committee approval has been obtained. The study was approved by the medical center’s institutional review board (Approval No. 2023 (1624)), and the requirement for informed patient consent was waived because of the retrospective observational study design.

#### 2.1.1. Inclusion Criteria

Patients with proven, probable, or possible IM, according to the definitions provided in Section 2.2, were included. Treatment was initiated with isavuconazole (six doses of 200 mg every 8 h, followed by 200 mg daily) or amphotericin B deoxycholate (0.5–0.7 mg/kg/day intravenously). The dosing of isavuconazole and amphotericin B deoxycholate was determined based on clinical guidelines and previous studies [16,19,20,24]. The initial treatment was maintained for a minimum of 3 days. All patients were aged ≥14 years.

#### 2.1.2. Exclusion Criteria

Patients were excluded if they had less than 60% of the data completed, were lost to follow-up, or if they were aged <14 years.

Comprehensive demographic and clinical data, including age, sex, obesity (according to body mass index [BMI]), hypertension, diabetes mellitus, underlying malignancy, history of allogeneic stem cell transplantation, presence of graft-versus-host disease, solid organ transplantation, history of corticosteroid and immunosuppressant use, and hepatic and renal disorders at admission, were collected for all participants. The infection sites, pathogens, types, and antifungal treatments for IM were recorded. Outcome information, including response to primary therapy, all-cause mortality, IM-attributable mortality, and adverse events, was also collected. The data were systematically collected using secure, standardized forms, and stored in an analytical file system (RedCap).

### 2.2. Diagnostic, Treatment, and Outcome Definitions

The revised 2020 European Organization for Research and Treatment of Cancer and the Mycoses Study Group Education and Research Consortium criteria were adopted as the diagnostic criteria for proven and probable IM [26]. Proven IM was defined as documented histopathological and microbiological evidence of mycosis infection on tissue biopsy or on a needle aspiration specimen from a typically sterile site (excluding bronchoalveolar lavage fluid, cranial sinus cavity, and urine) or by isolation of mycoses from blood. Probable IM was defined as the presence of at least one host factor (e.g., hematologic malignancy, receipt of an allogeneic stem cell transplant, receipt of a solid organ transplant, corticosteroid use, severe immunodeficiency, or acute graft-versus-host disease) along with one clinical criterion (e.g., pulmonary nodules, air crescent sign, cavity on computed tomography, tracheobronchitis, or sinonasal diseases) and one mycological criterion (e.g., recovery of *Mucorales* by culture or detection of mold elements in the sputum, bronchoalveolar lavage, bronchial brushings, or aspirate samples). For possible IM, the definition was revised based on previous literature [12,27]. Possible IM was characterized by the presence of a host factor and clinical criteria strongly suggestive of IM, where no alternative diagnosis was established, and treatment with antifungal therapy was decided upon by multidisciplinary team discussion.

Primary antifungal therapy was defined as the first therapy used upon diagnosis of IM. Salvage therapy was considered as any regimen administered after primary therapy. Based on the clinical and radiological outcomes, the therapeutic response was evaluated at week 6 after initiating the primary therapy.

The treatment responses were classified as follows: complete response (resolution of symptoms and radiological improvement), partial response (improvement in clinical and radiological findings but not fully resolved), stable (no clinical changes or minor radiological improvement), or failure (worsening of clinical and radiological findings). Additionally, in accordance with the ICH E9 Statistical Principles for Clinical Trials and previous clinical studies on the definition of treatment failure [28,29,30], patients who received salvage therapy due to adverse events related to the primary treatment were classified as having experienced treatment failure. Death was evaluated at week 12 after the diagnosis of IM.

### 2.3. Statistical Analysis

Categorical variables were compared using the chi-square test or Fisher’s exact test, as needed. Continuous variables were compared using one-way analysis of variance or the Mann–Whitney *U* test. If a significant result (*p* < 0.05) was detected for tests involving multiple group comparisons, pairwise comparisons were performed with the α level adjusted using Holm’s sequential Bonferroni adjustment to control for type I error. All tests were two-sided with a significance level of 0.05, except for the pairwise comparisons with α adjustment. Data analyses were performed using IBM SPSS Statistics for Windows (version 29.0).

## 3. Results

### 3.1. Demographic and Clinical Characteristics

**Figure 1:**
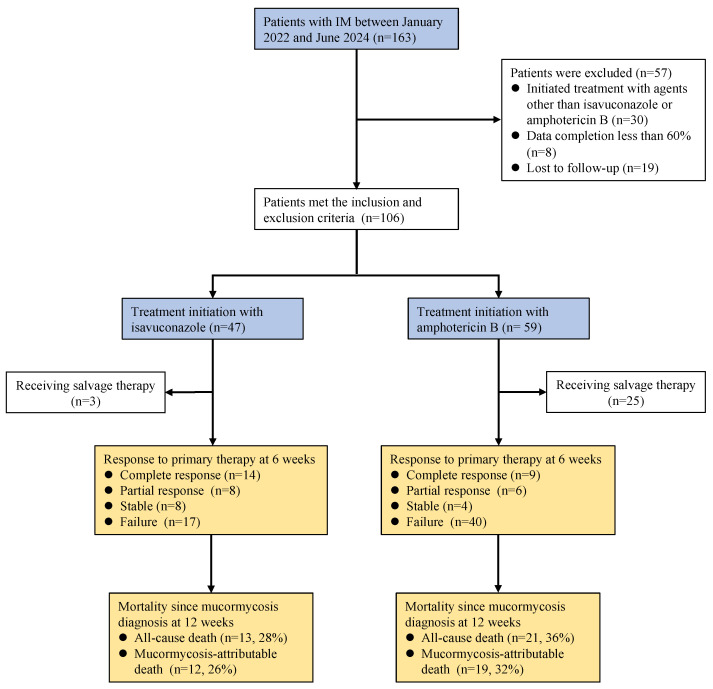
Flowchart of patients included and excluded from this study.

Among the 163 patients with IM screened at a comprehensive medical center in Western China from January 2022 to June 2024, 106 met the study criteria (Figure 1). We excluded 57 patients because treatment was initiated with agents other than isavuconazole or amphotericin B (n = 30); data completion was <60% (n = 8), and individuals were lost to follow-up (n = 19) (Figure 1).

Among the patients whose data were included in the analysis, the median age was 53 years (range 14–79 years), and the median BMI was 21.66 (15.56–35.75) kg/m^2^, with 69% of the patients (n = 73) being male (Table 1). The most prevalent underlying condition was diabetes mellitus (65%), followed by hypertension (30%), acute leukemia (15%), allogeneic stem cell transplantation (9%), solid organ transplantation (8%), and graft-versus-host disease (7%) (Table 1). A history of corticosteroid and immunosuppressant use was noted in 34% and 29% of the patients, respectively (Table 1). Before initiating antifungal therapy, 25% of the patients had renal disorders, and 18% had hepatic disorders (Table 1).

### 3.2. IM Infection and Therapy

For patients with IM, the most common site of infection was pulmonary (79%), affecting 84 patients, followed by disseminated (11%) and extrapulmonary (9%) infections (Table 1). The most frequently collected specimen was bronchoalveolar lavage fluid (55%), and next-generation sequencing was the predominant examination method (67%) (Table 1). The predominant pathogens associated with IM were *Rhizopus* spp. (41%), followed by *Rhizomucor* spp. (24%), *Lichtheimia* spp. (11%), *Mucor* spp. (8%) and *Cunninghamella* spp. (3%) (Table 1). Among all patients with IM included in the analysis, 23 patients (22%) were defined as having proven infection, 74 patients (70%) as having probable infection, and nine patients (8%) as having possible infection (Table 1). For primary antifungal therapy, 47 patients (44%) received isavuconazole, whereas 59 patients (56%) were treated with amphotericin B (Table 1).

The two groups of patients presented similar characteristics in terms of sex, BMI, diabetes mellitus status, malignant tumors, organ transplantation, graft-versus-host disease, site of infection, and pathogens (Table 2). Older patients with hypertension and renal disorders demonstrated a preference for isavuconazole as the primary antifungal therapy (Table 2). Because of drug-related adverse events or clinical effectiveness, the proportion of patients receiving salvage therapy was significantly greater in the amphotericin B group than in the isavuconazole group (42% vs. 6%, *p* < 0.001) (Table 2). Among the patients receiving salvage therapy, the median time to salvage therapy was 5 days in the isavuconazole group and 10 days in the amphotericin B group after the start of initial treatment (Table 2). In the amphotericin B group, 20 patients discontinued treatment and received antifungal therapy with isavuconazole (10 patients), posaconazole (7 patients), or liposomal amphotericin B (3 patients). Additionally, five patients were treated with other antifungal agents for IM in combination with amphotericin B (Table 2). In the isavuconazole group, no patients discontinued treatment, although three patients received other antifungal agents for IM in combination with isavuconazole (Table 2).

### 3.3. Adverse Events

Adverse events related to primary antifungal therapy occurred in 51% of the patients (54/106), with a notable difference between the treatment groups. Adverse events were reported in five patients in the isavuconazole group compared with 49 patients in the amphotericin B group (*p* < 0.001) (Table 1 and Table 2). No patients in the isavuconazole group discontinued treatment because of adverse events. By contrast, in the amphotericin B group, 17 patients discontinued treatment because of renal disorders, and one patient discontinued treatment because of gastrointestinal disorders. In the isavuconazole group, hepatic disorders were observed in four patients (9%), and a gastrointestinal disorder was observed in one patient (2%) (Table 2). For those in the amphotericin B group, the most common adverse events were hypokalemia (64%), followed by renal disorders (46%), hepatic disorders (17%), gastrointestinal disorders (7%), and neurologic disturbances (2%) (Table 2). The incidence of renal disorders and hypokalemia was notably greater in the amphotericin B group than in the isavuconazole group (*p* < 0.001 and *p* < 0.001, respectively) (Table 2).

### 3.4. Outcomes

Overall, IM showed a complete therapeutic response in 22% of the patients, a partial response in 13%, a stable response in 11%, and failure in 54% (Table 1). Patients treated with isavuconazole exhibited significantly superior therapeutic responses than those receiving amphotericin B (*p* = 0.013), with fewer treatment failures in the isavuconazole group (36% vs. 68%, *p* = 0.001) (Table 1). We found no significant differences in the rates of complete, partial, or stable responses between the two groups (Table 1). The overall all-cause mortality rate among the patients was 32%, with a mucormycosis-attributable mortality rate of 29%. Compared with the amphotericin B group, the isavuconazole group presented lower all-cause mortality (28% vs. 36%) and mucormycosis-attributable mortality (26% vs. 32%) rates. However, the differences were not statistically significant (Table 2).

## 4. Discussion

IM remains a substantial health threat, particularly for immunocompromised individuals. The therapeutic options for IM are limited, with amphotericin B often criticized for its adverse effects and requirement for intravenous administration. Despite advancements in liposomal formulations, these challenges persist [21]. Isavuconazole has emerged as a promising therapeutic option for IM, and has favorable effectiveness in clinical settings. The VITAL study suggested that the effectiveness of isavuconazole in treating IM is comparable to that of amphotericin B, with better tolerability reported [25]. However, since the approval of isavuconazole, there has been a lack of real-world studies focusing on its effectiveness and safety in the treatment of IM. While a few studies have compared the efficacy and safety of isavuconazole and amphotericin B in invasive fungal infections, most cases involved invasive aspergillosis rather than IM [31,32,33,34,35]. The limited number of IM cases included in these studies and the heterogeneity of their results highlight a critical knowledge gap regarding the real-world efficacy and safety of isavuconazole versus amphotericin B in treating IM [31,32,33,34,35]. To our knowledge, this study is the first real-world comparison of the effectiveness and safety of isavuconazole and amphotericin B, specifically for the treatment of IM.

Our cohort of patients with IM exhibited a high prevalence of underlying diseases, with diabetes mellitus, hypertension, and acute leukemia being the most common, which is consistent with previous literature [4,36]. Similar demographic characteristics, underlying diseases, and histories of corticosteroid and immunosuppressant use between the isavuconazole and amphotericin B groups provided a solid basis for subsequent evaluations of the effectiveness and safety profiles of these agents. Notably, patients with renal disorders demonstrated a preference for isavuconazole as the primary therapy, which is attributable to the unsuitability of amphotericin B for individuals with this condition. This trend aligns with the current clinical consensus [16,24].

In our study, the most common site of IM infection was the lungs, which differs from previous studies. Earlier research indicated that patients with diabetes mellitus are prone to rhino-orbito-cerebral mucormycosis [37]. This transition may be attributed to advancements and applications in molecular diagnostic techniques, which offer high sensitivity and specificity and are less time-consuming than traditional diagnostic methods [27,38,39]. The present study frequently used bronchoalveolar lavage fluid samples and next-generation sequencing for the diagnosis of IM. One challenge in using molecular diagnostics to diagnose pulmonary mucormycosis is differentiating colonizers from invasive infections [40]. To address this issue, we formed a multidisciplinary team to thoroughly discuss each case included in the analysis. The most prevalent pathogens were *Rhizopus* spp., which aligns with the epidemiological patterns observed in certain global regions, highlighting the diverse epidemiology of IM across different geographical areas [1,4,36].

Currently, the primary concerns with isavuconazole include hepatotoxicity, gastrointestinal disorders, and neurological toxicity [10,25]. In the isavuconazole treatment group, hepatic disorders occurred in four patients, and a gastrointestinal disorder occurred in one patient. Despite these events, the incidence of adverse effects has remained low and did not necessitate isavuconazole discontinuation. By contrast, amphotericin B treatment is associated with various toxicities, including nephrotoxicity, hepatotoxicity, and electrolyte imbalances [20,41]. In the present study, the participants in the amphotericin B group exhibited significant instances of renal disorders and hypokalemia, with 17 patients discontinuing amphotericin B because of renal disorders. The rates of adverse events related to hypokalemia and renal disorders were significantly greater in the amphotericin B group than in the isavuconazole group. These findings underscore the favorable safety profile of isavuconazole in the management of IM. Isavuconazole inhibits cytochrome P450 (CYP)-dependent 14α-lanosterol demethylation, disrupting fungal membrane ergosterol synthesis and ultimately causing fungal cell death [42]. Amphotericin B exerts its antifungal effects by binding directly to fungal cell membranes, inducing pore formation, increasing membrane permeability, and promoting oxidative damage [43]. The adverse effects associated with isavuconazole and amphotericin B are closely linked to their respective antifungal mechanisms, which may explain the better safety profile of isavuconazole in treating IM, at least in part.

In the present study, the isavuconazole group presented a lower incidence of treatment response failure than the amphotericin B group. Nevertheless, we found no significant difference between the two groups concerning the treatment response, which was classified as a complete response, a partial response, or stable. Notably, at week 6, the complete response rate for patients receiving isavuconazole was 30%, which was higher than the 11% clinical remission rate reported in the VITAL study, but lower than the 83.3% rate observed in other studies [25,31]. Isavuconazole demonstrates favorable bioavailability, excellent tissue penetration, fewer adverse effects, and a lower potential for drug–drug interactions [44]. No patients discontinued isavuconazole due to adverse drug reactions. By contrast, amphotericin B has been associated with significant nephrotoxicity and poor drug tolerability because of its antifungal mechanisms [45]. In our study, some patients discontinued amphotericin B because of intolerance. These factors may partially explain the better treatment responses observed with isavuconazole. Furthermore, we found low all-cause mortality and mucormycosis-attributable mortality rates in the isavuconazole group. Nevertheless, compared with those in the amphotericin B group, the differences were not significant. The 12-week all-cause and mucormycosis-attributable mortality rates in the isavuconazole group were 28% and 26%, respectively, similar to previous studies. For instance, Mario Fernández-Ruiz and colleagues reported a 12-week all-cause mortality rate of 32.1% and a mucormycosis-attributable mortality rate of 22.2% in patients treated with isavuconazole for invasive mold diseases [10]. In the amphotericin B group, the 12-week all-cause and mucormycosis-attributable mortality rates were 36% and 32%, respectively, aligning with previous findings. For example, Abi Manesh et al. reported a 3-month all-cause mortality rate of 37% for COVID-19-associated rhino-orbito-cerebral mucormycosis treated with amphotericin B [11], while Valliappan Muthu et al. found a 90-day mortality rate of 28.6% in patients with pulmonary mucormycosis [12]. Previous studies have evaluated the activity of isavuconazole and amphotericin B against *Mucorales* isolates in vitro, demonstrating that their minimum inhibitory concentration distributions are species-dependent [46]. Isavuconazole exhibits favorable activity against *Lichtheimia, Rhizopus*, and *Rhizomucor* species in vitro [44,47], and also the predominant *Mucorales* members identified in our study. These findings strongly support the conclusions of this research. Despite the constraints posed by limited data, these findings support the effectiveness of isavuconazole in the treatment of IM.

The present study has several limitations, including its retrospective observational design, constraint to a single comprehensive medical center, and assessments conducted at only two time points, which may not comprehensively reflect the clinical response at the end of treatment. Nevertheless, a significant strength of our study is that it represents the first report of a real-world investigation directly comparing the effectiveness and safety of isavuconazole with amphotericin B for the treatment of IM. To the best of our knowledge, this comparison has not been reported previously.

## 5. Conclusions

Isavuconazole demonstrated a favorable clinical response and safety profile when used as an antifungal treatment for patients with IM, outperforming amphotericin B in terms of both clinical efficacy and safety. Prospective clinical trials are needed to provide additional robust evidence and validate these findings.

## Figures and Tables

**Table 1:** Patients’ characteristics, treatment, and outcomes.

Variables	Patients (n = 106) N (%) ^†^
Age (years), median (range)	53 (14–79)
Sex, male	73 (69)
BMI, median (range)	21.66 (15.56–35.75)
**Underlying diseases**	
Hypertension	32 (30)
Diabetes	69 (65)
Acute leukemia	16 (15)
Lymphoma	3 (3)
Solid tumors	3 (3)
Allogenic stem cell transplantation	10 (9)
Solid organ transplantation	8 (8)
Graft-versus-host disease	7 (7)
Hepatic disorders ^‡^	19 (18)
Renal disorders ^‡^	26 (25)
Use of corticosteroids ^‡^	36 (34)
Use of immunosuppressants ^‡^	31 (29)
**Site of infection**	
Pulmonary	84 (79)
Extrapulmonary	10 (9)
Disseminated ^§^	12 (11)
**Specimens**	
Blood	8 (8)
Bronchoalveolar lavage fluid	55 (55)
Sputum	16 (16)
Biopsy tissue	12 (12)
Cerebrospinal fluid	4 (4)
Exudate	3 (3)
**Examination Methods**	
Culture	16 (16)
Next-Generation Sequencing	66 (67)
Pathology	16 (16)
**Organism of mucormycosis**	
*Cunninghamella* spp.	3 (3)
*Lichtheimia* spp.	11 (11)
*Mucor* spp.	8 (8)
*Rhizomucor* spp.	24 (24)
*Rhizopus* spp.	40 (41)
Order *Mucorales*	12 (12)
**Diagnosis of mucormycosis**	
Proven	23 (22)
Probable	74 (70)
Possible	9 (8)
**Primary therapy**	
Isavuconazole	47 (44)
Amphotericin B	59 (56)
**Adverse events related to primary therapy**	54 (51)
**Response to primary therapy at 6 weeks**	
Complete response	23 (22)
Partial response	14 (13)
Stable	12 (11)
Failure	57 (54)
**Mortality since mucormycosis diagnosis at 12 weeks**	
All-cause death	34 (32)
Mucormycosis-attributable death	31 (29)

^†^: For any variable with missing data, the number of patients with data available for this variable was added as the denominator. ^‡^: Before the diagnosis of invasive mucormycosis. ^§^: Two or more sites of infection with *Mucor*.

**Table 2:** Comparison of primary antifungal therapy.

Characteristics and Outcomes	Isavuconazole (n = 47) N (%) ^†^	Amphotericin B (n = 59) N (%) ^†^	*p*-Value
Age (years), median (range)	56 (22–79)	49(14–76)	**0.003**
Sex, male	35 (75)	38 (64)	0.266
BMI, median (range)	21.76 (15.56–30.49)	21.64 (15.61–35.74)	0.801
**Underlying diseases**			
Hypertension	19 (40)	13 (22)	**0.040**
Diabetes	29 (62)	40 (68)	0.513
Malignant tumors	9 (19)	13 (22)	0.716
Organ transplantation	7 (15)	11 (19)	0.609
Graft-versus-host disease	2 (4)	5 (9)	0.459
Hepatic disorders ^‡^	9 (19)	10 (17)	0.769
Renal disorders ^‡^	20 (43)	6 (10)	**<0.001**
Use of corticosteroids ^‡^	19 (40)	17 (29)	0.210
Use of immunosuppressants ^‡^	14 (30)	17 (29)	0.913
**Site of infection**			0.189
Pulmonary	41 (87)	43 (73)	
Extrapulmonary	3 (6)	7 (12)	
Disseminated	3 (6)	9 (15)	
**Organism of mucormycosis**			0.607
*Lichtheimia* spp.	6 (13)	5 (9)	
*Rhizomucor* spp.	8 (17)	16 (27)	
*Rhizopus* spp.	16 (34)	24 (41)	
Other *Mucorales*	11 (23)	12 (20)	
**Diagnosis of mucormycosis**			0.105
Proven	10 (21)	13 (22)	
Probable	30 (64)	44 (75)	
Possible	7 (15)	2 (3)	
**Receiving salvage therapy**	3 (6)	25 (42)	**<0.001**
Isavuconazole	0 (0)	10 (17)	**0.002**
Posaconazole	0 (0)	7 (12)	**0.017**
Liposomal amphotericin B	0 (0)	3 (5)	0.253
Combination therapy	3 (6)	5 (9)	1.000
**Time to initiation of salvage therapy (days), median**	5	10	0.457
**Adverse events related to primary therapy ^§^**	5 (11)	49 (83)	**<0.001**
Gastrointestinal disorders	1 (2)	4 (7)	0.379
Hepatic disorders	4 (9)	10 (17)	0.202
Renal disorders	0 (0)	27 (46)	**<0.001**
Hypokalemia	0 (0)	38 (64)	**<0.001**
Neurologic disturbances	0 (0)	1 (2)	1.000
**Response to primary therapy at 6 weeks**			**0.013**
Complete response	14 (30)	9 (15)	0.071
Partial response	8 (17)	6 (10)	0.301
Stable	8 (17)	4 (7)	0.098
Failure	17 (36)	40 (68)	**0.001**
**Mortality since mucormycosis diagnosis at 12 weeks**			
All-cause death	13 (28)	21 (36)	0.385
Mucormycosis-attributable death	12 (26)	19 (32)	0.453

^†^: For any variable with missing data, the number of patients with data available for this variable was added as the denominator. When the *p*-value was less than the significance level, it was highlighted in bold. ^‡^: Before the diagnosis of invasive mucormycosis. ^§^: Gastrointestinal disorders include nausea, vomiting, abdominal pain, or diarrhea. Hepatic disorders are characterized by the elevation of aspartate transaminase or alanine transaminase to more than twice the upper limit of normal, or total bilirubin, exceeding once the upper limit of normal. Renal disorders are defined as an estimated glomerular filtration rate of less than half the normal value, or creatinine greater than 200 μmol/L. Hypokalemia is indicated by a potassium concentration below 3.5 mEq/L. Neurologic disturbances include psychiatric or visual abnormalities.

## Data Availability

The original contributions presented in this study are included in the article. Further inquiries can be directed to the corresponding author.
